# The influence of knee position on ankle dorsiflexion - a biometric study

**DOI:** 10.1186/1471-2474-15-246

**Published:** 2014-07-23

**Authors:** Sebastian F Baumbach, Mareen Brumann, Jakob Binder, Wolf Mutschler, Markus Regauer, Hans Polzer

**Affiliations:** 1 Department of Trauma Surgery – Campus Innenstadt, Munich University Hospital, Nussbaumstr. 20, 80336 Munich, Germany

**Keywords:** Musculus gastrocnemius tightness, Knee flexion, Range of motion, Ankle joint

## Abstract

**Background:**

Musculus gastrocnemius tightness (MGT) can be diagnosed by comparing ankle dorsiflexion (ADF) with the knee extended and flexed. Although various measurement techniques exist, the degree of knee flexion needed to eliminate the effect of the gastrocnemius on ADF is still unknown. The aim of this study was to identify the minimal degree of knee flexion required to eliminate the restricting effect of the musculus gastrocnemius on ADF.

**Methods:**

Bilateral ADF of 20 asymptomatic volunteers aged 18-40 years (50% female) was assessed prospectively at six different degrees of knee flexion (0°, 20°, 30°, 45°, 60°, 75°, Lunge). Tests were performed following a standardized protocol, non weightbearing and weightbearing, by two observers. Statistics comprised of descriptive statistics, t-tests, repeated measurement ANOVA and ICC.

**Results:**

20 individuals with a mean age of 27 ± 4 years were tested. No significant side to side differences were observed. The average ADF [95% confidence interval] for non weightbearing was 4° [1°-8°] with the knee extended and 20° [16°-24°] for the knee 75° flexed. Mean weightbearing ADF was 25° [22°-28°] for the knee extended and 39° [36°-42°] for the knee 75° flexed. The mean differences between 20° knee flexion and full extension were 15° [12°-18°] non weightbearing and 13° [11°-16°] weightbearing. Significant differences of ADF were only found between full extension and 20° of knee flexion. Further knee flexion did not increase ADF.

**Conclusion:**

Knee flexion of 20° fully eliminates the ADF restraining effect of the gastrocnemius. This knowledge is essential to design a standardized clinical examination assessing MGT.

## Background

Various pathologies affecting the lower extremity, including plantar heel pain [1-3], metatarsalgia [4,5], stress fractures of the foot, and Achilles tendionpathy [6] are associated with limited ankle dorsiflexion (ADF). During gait reduced ADF results in an increase of forefoot pressure, which might be responsible for the above outlined pathologies [7-9]. Studies were able show, that increasing ADF in these patients leads to a reduction of the symptoms [10-13]. ADF can be impaired due to osseous, ligmentous, neurologic or muscular restrains, with musculus gastrocnemius tightness (MGT) being the most common cause [1,14].

**Figure 1:**
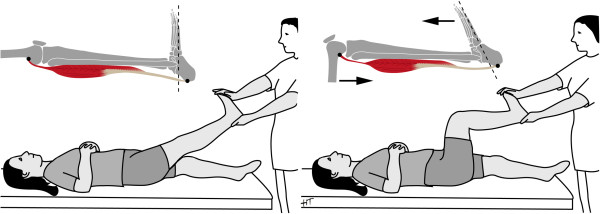
Schematic illustration of the anatomical and testing principles of the relation between ankle dorsiflexion and the knee position.

The musculus gastrocnemius has an influence on ADF because it bridges the knee and ankle joint. Under physiological conditions the gastrocnemius is under full tension when the knee is extended, as the muscle’s origin is furthest from its insertion. ADF is then restrained by the muscle’s tension. On the contrary, knee flexion increases ADF, as the muscle’s origin and insertion are approximated. Further ADF is then limited by other structures of the ankle joint (Figure 1). In symptomatic patients the first assessment should therefore evaluate ADF with the knee fully extended. In case of impaired ADF one should then identify whether ADF can be increased by knee flexion. Patients with MGT demonstrate a reduced ADF with the knee fully extended, but ADF can be increased by knee flexion [2]. Identification of isolated MGT is essential for both physiotherapists and physicians, as it can be treated by stretching or endoscopic musculus gastrocnemius recession. If ADF cannot be increased by knee flexion the gastrocnemius is not responsible for the impaired ADF.

Consequently, any clinical test investigating MGT compares ADF with the knee extended to ADF with the knee flexed. This procedure was first described in 1923 by Nils Otto Silfverskiöld, an Swedish orthopedic surgeon [15].

In the literature, numerous tests are described to assess ADF. These tests can be categorized into non weightbearing [3,16], weightbearing [17,18], and instrumented [19,20]. In everyday practice non weightbearing measurements are most commonly performed [21]. Nevertheless, evidence suggests a higher intra- and interrater reliability for weightbearing compared to non weightbearing measurements [17,18,21-23]. Furthermore, maximum ADF significantly differs from weightbearing to non weightbearing [23].

Independent of the test applied, no standard has been defined for the degree of knee flexion needed to eliminate the effect of the musculus gastrocnemius on ADF. Most studies conducting non weightbearing measurements applied a knee flexion of 90° [3,7,16]. On the contrary, most weightbearing measurements do not control for knee flexion [16,23]. Furthermore, weightbearing measurements with 90° knee flexion are not feasible. Consequently, it is of upmost importance to identify the minimal required degree of knee flexion to eliminate the effect of the musculus gastrocnemius on ADF.

To our best knowledge the degree of knee flexion needed to eliminate the restraining effect of the gastrocnemius on ADF is unknown. Therefore, the aim of this study was to identify the influence of varying amounts of knee joint flexion on ADF.

## Methods

### Study design and population

The study was approved by the local ethics committee of the University of Munich (# 007-14). Both ankles of 20 healthy individuals, aged 18 - 40 years, 50% female, were tested according to a standardized protocol, following screening and informed consent. The inclusion and exclusion criteria are presented in Table 1.

**Table 1:** Inclusion and exclusion criteria

**Inclusion criteria**	**Exclusion criteria**
Age: 18 to 40 years	Prior injuries to the knee, ankle or foot
Informed consent	Knee, ankle or foot pain within the last 2 years
Subjects can read and understand German	History of ankle trauma or surgery
	Impaired knee range of motion
	Conditions/systematic diseases possibly affecting the neuromuscular or musculoskeletal system
	Pregnancy [anamnestic]
	Cardiovascular diseases including thrombosis
	Regular medication excluding contraceptives
	Subject is unable to give informed consent

### Measurement procedure

**Figure 2:**
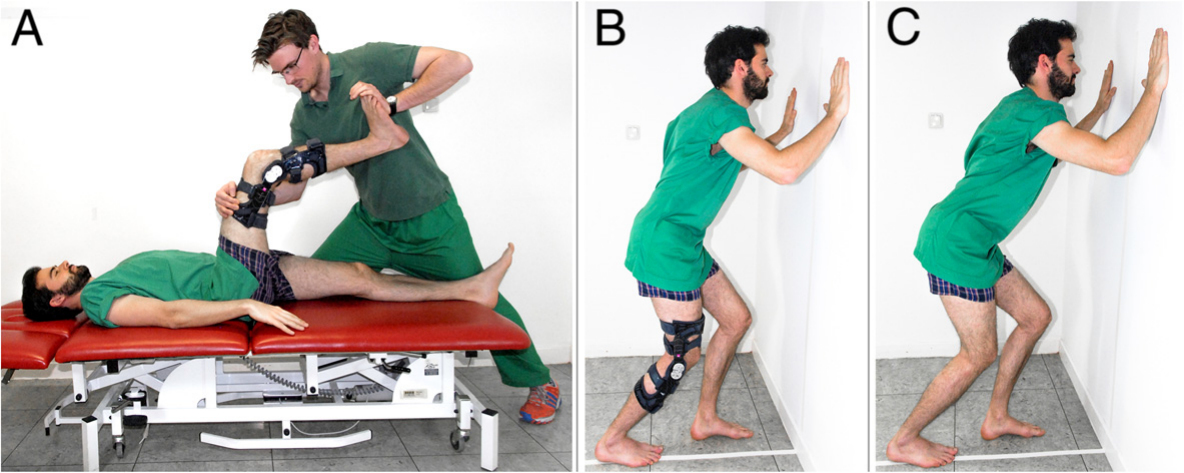
**Exemplary test procedure. A)** Non weightbearing measurement; **B)** Weightbearing measurement; **C)** Lunge test; The image was taken by the authors and the shown persons gave informed consent for publishing their image.

ADF measurements were conducted both weightbearing and non weightbearing at different degrees of knee flexion and in a lunge position, following a standardized protocol. Each measurement was performed by two investigators (SFB, HP), blinded to each other’s results. A standard goniometer (MDF Instruments USA, Inc. Malibu, CA, USA) with 2° increments and 20 cm length was used. Anatomical measurement landmarks were the long axis of the fibula and the fifth metatarsal bone [16,24-27], which were marked prior to testing [24,28]. A functional brace (Medi M4, Medi GmbH & Co. KG, Bayreuth, Germany), which fixes the knee in various angles, was used to control the knee position. ADF was assessed at the following degrees of knee flexion: full extension, 20°, 30°, 45°, 60° and 75°. The order of examiners and knee flexion (full extension to 75° vs. 75° to full extension) were altered between the subjects. The subjects rested for 30 seconds between measurements.Non weightbearing ADF measurements were taken with the individuals in supine position. One investigator applied maximum ADF with the foot in subtalar neutral position, while the other performed the measurement (Figure 2A; the shown persons gave informed consent for publishing their image).For weightbearing ADF measurements, the subject was asked to stand in a lunge position with the back leg being the one measured. The second toe and heel were centered over a line perpendicular to the wall. Patients were allowed to stabilize their stance by holding onto the wall. For the full dorsiflexion measurements, the patient was then asked to lean forward just before heel lift off. One examiner assured maximum knee extension. For the other measurements, the subject was asked to flex their knee until fully restrained by the functional brace and then move their knee forward above the line just before heel lift off. The other examiner assured subtalar neutral position (Figure 2B).The final test performed was the Lunge test. The subject was asked to do a lunge with the back leg flexed and squad with the rear leg until just before the heel lifts off the ground. The second toe and heel were again centered over a line perpendicular to the wall. Patients were allowed to stabilize their stance by holding onto the wall. Subtalar neutral position was monitored (Figure 2C). The references used for all weightbearing measurements were the long axis of fibula and the ground.

#### Outcome variables and statistics

Data assessed were standard demographics, level of sports, dominant leg (defined as leg used to kick a ball), and maximum ADF. Values are presented as mean values ± standard deviations or the 95% confidence intervals (CI).

A sample size calculation could not be performed due to missing preliminary data. The sample size was chosen based on previous studies on MGT [2,18,21]. Mann-Whitney-U-Tests were used to compare gender-, side-, and non-/weightbearing differences. A Bonferroni correction was used to account for multiple testing (p < 0.004). A repeated measurement ANOVA was conducted to assess significant differences for ADF between the different degrees of knee flexion. Test reliability was assessed by interrater reliability using the interclass correlation coefficient (ICC). Interrater reliability defines the consistency of a measure taken by two examiners. ICC values range from 0 to 1, with 1 being perfect agreement. In general, an ICC greater than 0.7 is considered an acceptable level of reliability for clinical measures [29,30]. If not stated differently the values stated are the mean values of both investigators. Statistics were computed using SPSS Vs. 21 (IBM Company).

## Results

### Participant characteristics

Twenty healthy individuals (50% female) with a mean age of 27.1 ± 3.9 years (height: 175.0 ± 9.8 cm; weight: 68.7 ± 10.9 kg) were examined. The dominant foot, defined as the foot used to kick a ball was in all but one case the right foot. All but one participant (no sport) indicated that they participate in sporting activities 2-3 times per week. Statistical assumptions for normal distribution were not met (D’Agostino and Pearson Test). Table 2 shows the descriptive statistics for ADF for each degree of knee flexion separately.

**Table 2:** Degrees of ankle dorsiflexion at various degrees of knee flexion

**Knee flexion**	**Full Ext.**	**20°**	**30°**	**45°**	**60°**	**75°**	**Lunge**
NWB right	4 ± 8	20 ± 9	21 ± 9	21 ± 9	21 ± 9	21 ± 9	
WB right	25 ± 7	39 ± 7	39 ± 7	39 ± 7	39 ± 7	39 ± 7	40 ± 7
NWB left	4 ± 7	18 ± 7	19 ± 9	20 ± 10	20 ± 10	20 ± 9	
WB left	24 ± 6	37 ± 6	38 ± 7	38 ± 6	38 ± 7	38 ± 7	39 ± 7

Values given as mean ± standard deviation; **°**: degrees; NWB: non weightbearing; WB: weightbearing; Full Ext.: Full extension.

### Reproducability analysis

The interrater ICC ranged from 0.971 to 0.988 for non weightbearing, and from 0.961 to 0.992 for weightbearing measurements.

### Inferential analysis

**Figure 3:**
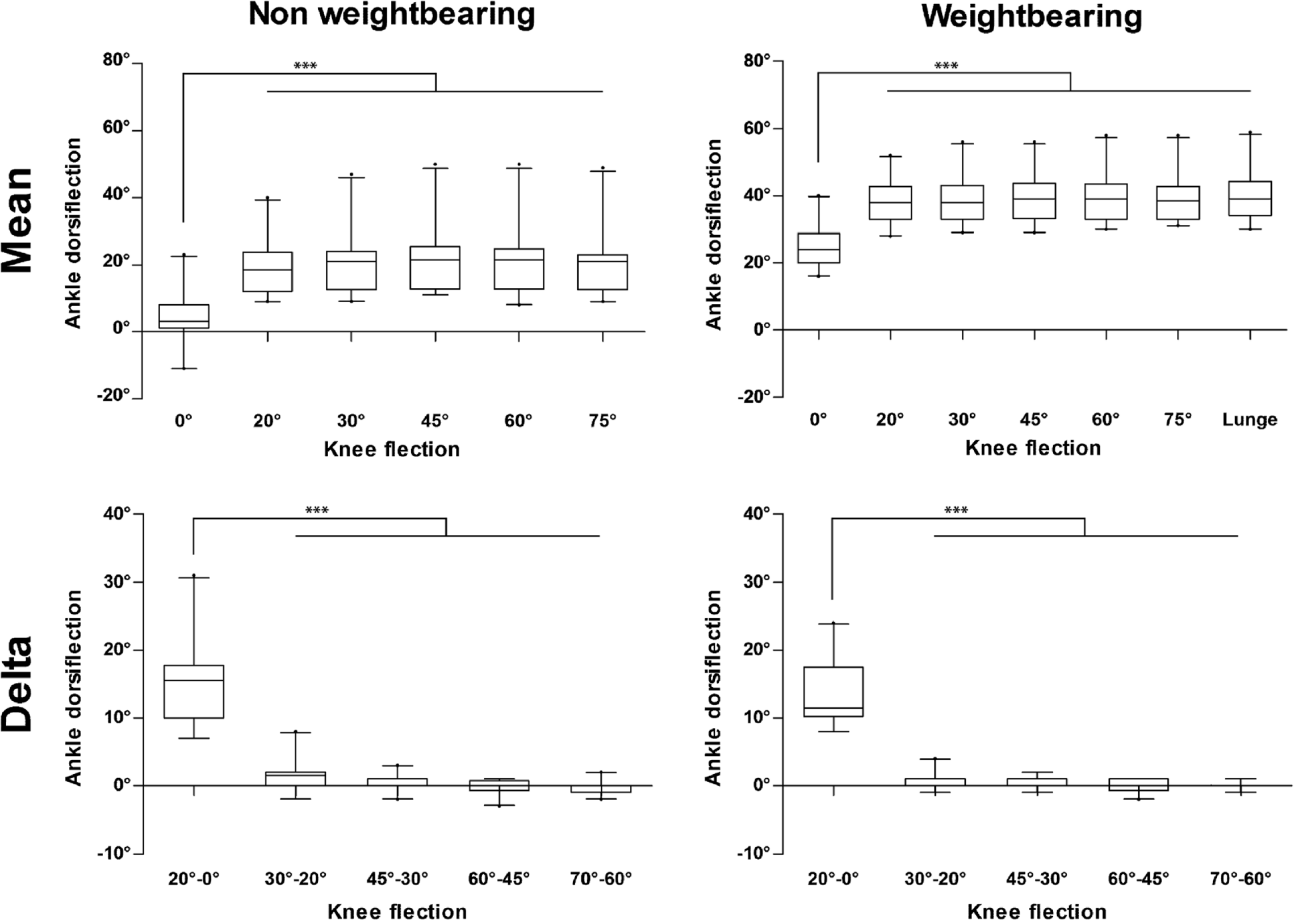
**Pooled data for mean ankle dorsiflexion and the mean differences between each increment of knee flexion presented as a box plot.** Mean: Pooled mean values for ankle dorsiflexion; Dela: Differences between each step of knee flexion; ***: p < 0.001.

No significant gender differences could be found for any measurement. A Mann-Whitney-U-Test revealed no significant differences between the right and left leg for neither non weightbearing nor weightbearing measurements. Pooled mean ADF (mean values of the right and left ankle) for the extended knee non weightbearing were 4° ± 7° (95% CI: 1°-8°; range: 11° - 23°) and weightbearing 25° ± 6° (95% CI: 22°-28°; range: 16° - 40°). For the knee 75° flexed non weightbearing values were 20 ± 9° (95% CI: 16°-24°; range: 9° - 49°) and weightbearing 39° ± 7° (95% CI: 36°-42°; range: 31° - 58°). The pooled data for non weightbearing and weightbearing ADF measurements and the differences between each step of knee flexion (delta) are presented in Figure 3. Weightbearing measurements were significantly greater than non weightbearing values (Mann- Whitney-U-Test, p < 0.001).

A repeated measurement ANOVA revealed significant differences (p < 0.001) between 0° and all other degrees of knee flexion (20°, 30°, 45°, 60°, 75°, Lunge). No significant differences for ADF could be observed when comparing 20° to 75° of knee flexion including the Lunge test. Consequently, knee flexion beyond 20° could not further increase ADF.

The difference for ADF (delta) between 20° knee flexion and full extension was 15° ± 6° [CI: 12° - 18°] for non weightbearing and 13° ± 5° [CI: 11° - 16°] for weightbearing ADF, which did not differ significantly. Starting from 20° knee flexion the maximum delta value observed was 1° [CI: 0° - 3°] for non weightbearing and 1° [CI: 0° - 1°] for weightbearing.

## Discussion

Musculus gastrocnemius tightness (MGT) is the most common cause for impaired dorsiflexion of the ankle joint [1,14]. It is held responsible for various pathologies affecting the lower extremity [4-6]. Identification of this cause by the treating physiotherapist or physician is extremely important as it can be easily treated by gastrocnemius lengthening either through physiotherapy or surgery. Various tests try to assess MGT by comparing ADF with the knee extended and flexed [3,18,20]. None of these tests has identified the degree of knee flexion needed to eliminate the effect of the gastrocnemius on ADF. Our study is the first to investigate the influence of the knee’s position on ADF. By 20° of knee flexion, the ADF restraining effect of a tight gastrocnemius on was already eliminated such that further knee flexion provided no additional ADF.

In the literature, different anatomical landmarks are described to assess the range of motion of the ankle joint. The landmarks most frequently used are the long axis of the fibula and either the plantar surface of the foot [21,31-33] or the axis of the fifth metatarsal bone [16,24-27,34-36]. During pretests, we found the accurate identification of the plantar aspect of the foot to be highly dependent on the hand position of the examiner. Therefore, we chose the long axis of the fibula and the fifth metatarsal bone as references for non weightbearing measurements. For weightbearing tests, the floor clearly defined the x-axis, but the long axis of the fibula was hard to be identified due to the prominence of the peroneal tendons while standing. We therefore decided to mark the long axis of the fibula and the fifth metatarsal bone prior to testing [24,28]. This procedure was also employed by Astroem and Arvidson [28] who conducted range of motion measurements to the foot in 121 healthy subjects. One has to keep in mind, that this might have a positive impact on the interrater reliability. In our study the interrater ICC values ranged from 0.961 to 0.992. These values are excellent, especially when compared to other non weightbearing measurements, with values ranging between 0.29 [36] and 0.81 [37]. Comparably good values have only been reported for weightbearing ADF measurements [17,38]. Our goal was to clearly define the degree of knee flexion needed to eliminate the effect of the musculus gastrocnemius on ankle dorsiflexion. Consequently, it was crucial to use a reliable measurement technique. The herein observed interclass correlation coefficient argues for our standardized measurement protocol.

We observed a great variation of ADF between the subjects with values ranging for the extended knee from -11° to 23° non weightbearing and from 16° to 40° weightbearing. For the knee 75° flexed values ranged from 9° to 49° non weightbearing and from 31° to 58° weightbearing. Comparable variations for ADF have been reported in the literature. Non weightbearing values range from -2° ± 5° [16] to 20° ± 5° [3] for the knee extended and from 12° ± 6° [16] to 25° ± 5° [3] for the knee flexed. Furthermore, we could observe significant differences between weightbearing and non weightbearing ADF measurements. This goes well in line with the observation of other authors [2,16,18,21,23,28]. Although there is less data available for weightbearing measurements, reported values range from 21° ± 7° [21] to 39° ± 5° [18] for the knee extended and from 33° ± 7° [16] to 50° ± 6° [23] for the knee flexed. The great range of ADF values might be due to the heterogeneity of the measurement procedures and the different landmarks used. Another pitfall we observed during the pretests was that minimal knee flexion had a profound impact on the ADF when measuring the ADF with the knee extended. Consequently, for our testing procedure one observer ensured full extension of the knee during measurements. Moreover, the great range of ADF might simply reflect the constitutional variation within the population.

Limitations that should be discussed are adjacent joint movements affecting ADF and the measurement device used. ADF is not limited to the tibiotalar joint, but also occurs partially in the subtalar and midtarsal joints. We tried to account for this problem by maintaining the foot in a subtalar neutral position, as recommended by previous authors [39-42]. Second, the goniometer used has 2° increments. Although other devices might be more accurate, the goniometer has to be considered the clinical gold standard [30]. With respect to the high ICC observed we believe this tool to be sufficiently accurate. Furthermore, because 20 degrees was the smallest knee flexion angle tested after full knee extension, it might be possible that even less knee flexion is already sufficient.. Nevertheless, we believe that every patient is capable to perform a Lunge test with 20° of knee flexion.

As stated above decreased ADF is held responsible for a variety of disorders. Up to date, there is neither a consensus on the degree of ADF considered pathological, nor whether tests should be conducted non weightbearing or weightbearing. The latter though has a pronounce impact on the degree of ADF measured. Weightbearing measurements have several advantages. First, they can be conducted by a single investigator. Second, they are independent of the torque applied by the observer, being a possible source of bias, and in addition more closely reflect the physiological torque during gait. Third, they have been shown to be more reliable [17,18,21-23]. Considering these aspects weightbearing tests should become the clinical standard. Nevertheless, most patients cannot conduct weightbearing tests with the knee 90° flexed, as conducted in non weightbearing measurements [3,7,16]. Consequently, it is of upmost importance to identify the minimal degree of knee flexion needed to eliminate the ADF restraining effect of the musculus gastrocnemius. This study is the first to investigate this problem. We were able to clearly demonstrate that already 20° of knee flexion sufficiently eliminates the effect of the musculus gastrocnemius on ADF.

## Conclusion

When assessing MGT the degree of knee flexion needed to eliminate the restraining effect of the musculus gastrocnemius on ADF was unknown. We were able to demonstrate that already 20° of knee flexion fully eliminates the restraining effect of the musculus gastrocnemius on ADF, both non weightbearing and weightbearing. Our results build the bases to define a standardized clinical examination for musculus gastrocnemius tightness.

## Abbreviations

ADF: Ankle dorsiflexion; MGT: Musculus gastrocnemius tightness; %: Percent; ICC: Interclass correlation coefficient; °: Degrees; #: Number; Cm: Centimeters; Kg: Kilograms.

## Competing interests

The authors declare that they have no competing interests.

## Authors’ contributions

SFB was involved in developing the study design, data acquisition and analysis and prepared the manuscript. MB reviewed the test setup and participated in the data acquisition. JB was responsible for subject acquisition, time management and test implementation. WM was involved in developing the study design, was an essential part of data interpretation. MR helped design the test setup acquire the data and prepared parts of the manuscript. HP had the study idea, was an essential party of data acquisition and interpretation and prepared wide parts of the manuscript. All authors read and approved the final manuscript.

## Pre-publication history

The pre-publication history for this paper can be accessed here:

http://www.biomedcentral.com/1471-2474/15/246/prepub
